# Peer Review Week 2022: an interview with Rafal Marszalek, Si Ming Man and Guideng Li about their views on research integrity as scientists and editors

**DOI:** 10.1038/s42003-022-03957-x

**Published:** 2022-09-20

## Abstract

Peer Review Week celebrates the essential role of peer review in maintaining the quality and integrity of science. This year’s theme is “Research Integrity: Creating and supporting trust in research.” In honour of this, here at *Communications Biology*, we spoke to Rafal Marszalek, Si Ming Man and Guideng Li about their views on research integrity as scientists and editors.

**Dr. Rafal Marszalek is the Chief Editor of** ***Scientific Reports*, an open access peer-reviewed journal.**

**Figure:**
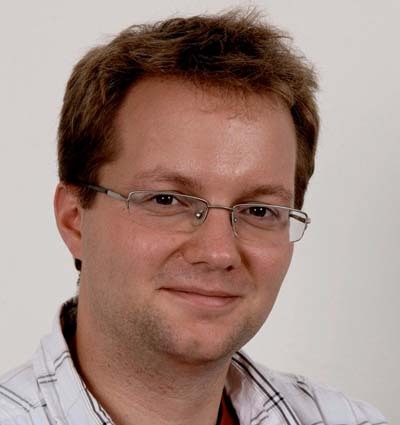
Rafal Marszalek

**Dr. Si Ming Man is a Professor at the Australian National University and an Editorial Board Member at *Communications Biology***.

**Figure:**
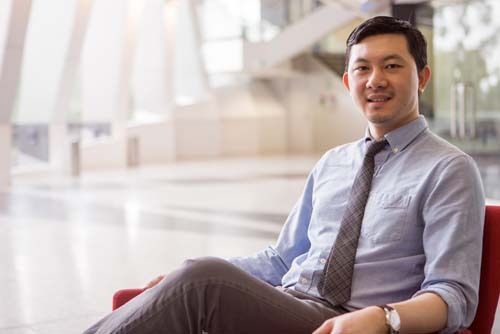
Si Ming Man

**Dr. Guideng Li is a Principal Investigator at the Institute of Systems Medicine/Suzhou Institute of Systems Medicine, Chinese Academy of Medical Sciences and an Editorial Board Member at** ***Communications Biology***.

**Figure:**
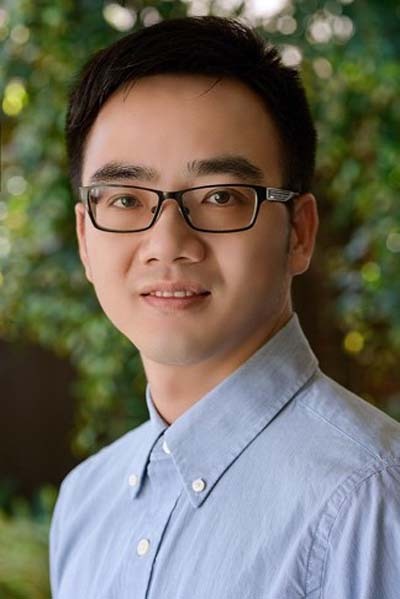
Guideng Li

Please tell us about your background and current position?

**[RM]:** I consider myself a biological and analytical chemist by training – my research focused on the development of methods for single cell proteomics applications. I became an editor 10 years ago – at BMC’s *Genome Biology* at first, where I handled research on cancer genomics, single cell omics, and gene editing.

In 2016 I moved to *Scientific Reports* and became Chief Editor of the journal in early 2022. I lead a team of in-house editors whose primary role is to provide support to our expert Editorial Board, enabling them to focus on the service to their communities - specifically helping peers evaluate and improve research, and disseminate findings quickly and without prejudice. We are also responsible for development and implementation of editorial and publishing policies, as well as handling of post-publication complaints, be it about the integrity of the work or a different issue.

**[SMM]:** My research interests are in the area of immunology, with the goal of understanding how innate immune responses are triggered during infectious disease and cancer. In addition to my role as Professor at The Australian National University, I am also an Editorial Board Member for *Communications Biology*, where I handle manuscripts across all areas of immunology and infectious disease. I am also the Immunology Section Lead for this journal.

**[GL]:** I am currently a Principal Investigator at the Institute of Systems Medicine/Suzhou Institute of Systems Medicine, Chinese Academy of Medical Sciences. I received my Ph.D. at the College of Medicine, University of California, Irvine in 2012 and then completed my postdoc training in Nobel laureate Dr. David Baltimore’s lab at the California Institute of Technology in 2019. I am serving as an Editorial Board Member for *Communications Biology*. My current research focuses on deciphering the molecular mechanisms responsible for productive or defective immune responses against cancer and developing new technologies for effective therapeutic strategies with potential clinical applications.

In your opinion, why is research integrity so important and what damage does research misconduct do to the field?

**[RM]:** I think something that can perhaps be easy to overlook when you are working on your own project within the confines of a laboratory environment is that science is a collective social enterprise, and the dissemination of findings – which is becoming increasingly easier – is a big part of it. And so it carries a lot of responsibility not just to perform research honestly and rigorously but to also communicate it transparently and without bias. The impact of research papers can be far-reaching: a single paper can influence clinical practice, can affect public policies (health, economic, environmental – take your pick), can be the basis for safety standards and so on. Fraud, misconduct, or just questionable practices can have two immediate serious impacts. Let me give you examples.

First, they can have a major influence on policies and practices. We don’t have to search far: the pandemic has provided us with ample examples. One would be that of hydroxychloroquine, a drug for malaria, lupus, and a few other conditions, which was tested for COVID in early 2020 in Gautret et al. The findings of this paper have since been quite thoroughly debunked, but not before they spurred more, sometimes questionable, research and clinical trials, and not before public figures’ support for the use of this drug for COVID most likely led to the buying spree and thence shortages of hydroxychloroquine in clinical settings where it is actually designed to be used. In some countries the use of hydroxychloroquine was promoted by authorities, becoming effectively a public health policy by fact, if not by name. It seems fairly clear now that it never was the wonder drug it was promised to be. That is not to say that there should not have been a study exploring it as a possibility though. But had such a study been designed properly, it would have likely shown what later studies did: that hydroxychloroquine has little clinical benefit in COVID treatment. And we can only speculate now on the number of lives this could have saved or the trials that could have been run instead to explore more realistic therapeutic options.

Second, breaches of research integrity can lead to a huge waste of money and resources. We tend to think of scientific research and pursuit of discovery as a higher goal, but no matter what value you put on knowledge, it does not change the fact that all research costs money and nobody has a bottomless pot of gold to pay for it. And so if a fraudulent or otherwise inaccurate study is published and leads to money being given to pursue lines of inquiry that may stem from these (potentially untrue) findings, this is precious funding that is not going elsewhere when it could. Even if fraud is uncovered and happens in a country that has regulations that allow the government to at least attempt to recuperate some of the money (such as the False Claim Act in the US), this can take a very long time. Now imagine that the research that did not get the grant because it had to compete with the fraudulent application is for a promising drug for some debilitating disease. Not being able to do this research is about more than researchers’ curiosity or even careers – it may mean life or death for many patients.

**[SMM]:** Research integrity is of the highest importance in science because it forms the basis of creating sound and reliable scientific knowledge. Research misconduct is damaging because it compromises trust in science and delays research progress, and at the same time, wastes valuable time and resources.

**[GL]:** I think research integrity becomes more and more important as expanding research findings have been reported. Researchers must be able to trust each other’s work, which would help speed up and also increase the quality of research.

As an active researcher, what do you do individually and what practices and values are important in your lab to support integrity?

**[SMM]:** I encourage my lab members to share their data with each other to facilitate a broad scientific discussion. We value open data discussion and believe this is critical for supporting research integrity. As a group leader, I have created an environment to support research integrity in the lab and to help minimise potential errors in data generation and presentation. Specifically, I regularly conduct reviews, in the form of a weekly data report, experimental planning, data analysis and data presentation of lab members. Senior members within the laboratory review data generated and analysed by junior researchers in the laboratory as part of their training to become future scientific leaders. We also interrogate data presented during lab meetings to ensure the highest levels of integrity. We strive to perform experiments independently by two or more researchers to ensure reproducibility where feasible. Multiple experimental approaches are used as much as practical to validate findings. In addition, data presentation is also cross-checked by several lab members before a manuscript is submitted.

Within our own department, we hold weekly ‘research in progress’ meetings where researchers share and discuss raw data to explore different interpretations, adding an extra layer of support to encourage data transparency. Our University holds research integrity workshops annually to ensure that our researchers understand and follow the best practices in their respective fields. I also think journals within the Springer Nature family, such as *Communications Biology*, provide an extra layer of support by asking authors to complete a manuscript checklist, which is seen by editors, peer reviewers, and the scientific community.

**[GL]:** I think most students, particularly first-year graduate students, have a vague understanding of general concepts related to research integrity. Thus, it is important for me to teach them research ethics and research integrity in a group setting and in one-on-one meetings. Also, I think it is important to remind them that the purpose of research is to discover something rather than to finish something.

As an external editor who is also an active researcher, how do you help to support research integrity in the manuscripts that you handle?

**[SMM]:** There are several stages in which an editor can support research integrity. As a manuscript proceeds through our peer review process, potential issues related to research integrity are discussed with in-house editors. In some cases, I have consulted with external peer reviewers asking for their comments and/or advice on papers suspected of a breach in research integrity. I would also encourage our reviewers to raise their concerns in the ‘Comments to the editors’ section, a confidential section only seen by the editors, if they happened to have identified issues related to research integrity. Sometimes the issues can be resolved by consulting with the authors and asking for their raw data.

**[GL]:** When I handle a manuscript, I will not only focus on the novelty of the study but also spend time checking if this manuscript has any research integrity issues, e.g., data fabrication, data falsification, and plagiarism.

Rafal, as a Chief Editor, what practices and values are important to your journal to ensure rigour?

**[RM]:** At *Scientific Reports* we work to develop workflows that help us identify problematic submissions readily, and rely on all sorts of checks using automated and semi-automated tools. Not everything can be detected with algorithms though: we must still rely on expert judgement about whether the study design is appropriate, whether the interpretation of the data makes sense, or what the quality of that data is. This is key here: we need people to conduct and evaluate research and to disseminate knowledge. We also need to remember that knowledge is a shared responsibility. And so one of the most important values in the publishing process is mutual trust. We have to trust that researchers did what they say they did. We have to trust that editors or reviewers declare their biases and conflicts. We have to trust that participants in the process fulfil their roles to the best of their abilities. And we have to trust that everyone is behaving ethically. This is perhaps another big way in which misconduct can damage science: it erodes the trust in the process.

I am of course not naïve enough to believe that trusting each other will ensure no research integrity problems. The opposite in fact: I don’t think there is a scientist out there who does not have their own agenda or incentives that drive them. Some of these may be more obvious than others, some may be more noble than others, and some may be more pernicious than others. What this means is that our trust can, and perhaps should, be accompanied by healthy skepticism. More importantly, it means that editors need to be open to the possibility of being wrong and need to be willing to act, at least to a degree to which it is possible within the procedural and legal constraints.

That being said, we cannot approach every paper with the baseline assumption that it is badly done or fraudulent. It is very simple: people do not go into research with the intention to deceive others. And so we just need to remember what the end goal is: it is to learn more about the world we live in. This is a major motivation for me as an editor but also a reason I love working at *Scientific Reports*–we have the ability to put the weirdest and yet most wonderful piece of knowledge in the hands of a curious child somewhere out there, and to inspire them to do something that will change the world one day. And if such knowledge is our hope for the future–why would you cheat about it?

*This interview was conducted by Zhijuan Qiu for Peer Review Week 2022*.

